# Impact of hydrogel peri-rectal spacer insertion on prostate gland intra-fraction motion during 1.5 T MR-guided stereotactic body radiotherapy

**DOI:** 10.1186/s13014-020-01622-3

**Published:** 2020-07-22

**Authors:** Francesco Cuccia, Rosario Mazzola, Luca Nicosia, Vanessa Figlia, Niccolò Giaj-Levra, Francesco Ricchetti, Michele Rigo, Claudio Vitale, Beatrice Mantoan, Antonio De Simone, Gianluisa Sicignano, Ruggero Ruggieri, Stefano Cavalleri, Filippo Alongi

**Affiliations:** 1 Advanced Radiation Oncology Deparment, Sacro Cuore Don Calabria Hospital, Negrar, Verona, Italy; 2 Urology Division, Sacro Cuore Don Calabria Hospital, Negrar, Verona, Italy; 3 University of Brescia, Brescia, Italy

**Keywords:** Prostate cancer, Stereotactic body radiotherapy, Organ motion, Mri-linac

## Abstract

**Background:**

The assessment of organ motion is a crucial feature for prostate stereotactic body radiotherapy (SBRT). Rectal spacer may represent a helpful device in order to outdistance rectal wall from clinical target, but its impact on organ motion is still a matter of debate. MRI-Linac is a new frontier in radiation oncology as it allows a superior visualization of the real-time anatomy of the patient and the current highest level of adaptive radiotherapy.

**Methods:**

We present data regarding a total of 100 fractions in 20 patients who underwent MRI-guided prostate SBRT for low-to-intermediate risk prostate cancer with or without spacer. Translational and rotational shifts were computed on the pre- and post-treatment MRI acquisitions referring to the delivery position for antero-posterior, latero-lateral and cranio-caudal direction, and assessed using the Mann-Whitney U-Test.

**Results:**

All patients were treated with a five sessions schedule (35 Gy/5fx) using MRI-Linac for a median fraction treatment time of 50 min (range, 46–65). In the entire study sample, median rotational displacement was 0.1° in cranio-caudal, − 0.002° in latero-lateral and 0.01° in antero-posterior direction; median translational shift was 0.11 mm in cranio-caudal, − 0.24 mm in latero-lateral and − 0.22 mm in antero-posterior. A significant difference between spacer and no-spacer patients in terms of rotational shifts in the antero-posterior direction (*p* = 0.033) was observed; also for translational shifts a positive trend was detected in antero-posterior direction (*p* = 0.07), although with no statistical significance. We observed statistically significant differences in the pre-treatment planning phase in favor of the spacer cohort for several rectum dose constraints: rectum V32Gy < 5% (*p* = 0.001), V28 Gy < 10% (*p* = 0.001) and V18Gy < 35% (*p* = 0.039). Also for bladder V35 Gy < 1 cc, the use of spacer provided a dosimetric advantage compared to the no-spacer subpopulation (*p* = 0.04). Furthermore, PTV V33.2Gy > 95% was higher in the spacer cohort compared to the no-spacer one (*p* = 0.036).

**Conclusion:**

In our experience, the application of rectal hydrogel spacer for prostate SBRT resulted in a significant impact on rotational antero-posterior shifts contributing to limit prostate intra-fraction motion. Further studies with larger sample size and longer follow-up are required to confirm this ideally favorable effect and to assess any potential impact on clinical outcomes.

## Introduction

The use of hypofractionated radiotherapy for prostate cancer has globally widespread, being endorsed by international guidelines [1, 2]. The radiobiological rationale for using higher doses per fraction in prostate cancer lies on the known low alpha-beta ratio of the tumor, estimated in 1.5 Gy, which reflects a superior sensitivity to > 2 Gy/fractions in terms of biological effect [3].

In recent years, technological progress has allowed clinicians to deliver very high doses per fraction with higher levels of accuracy and confidence [4].

The adoption of extremely hypofractionated schedules is gaining attention in the scientific community, as a fast and effective treatment option for PC patients [5–8]. In fact, stereotactic body radiotherapy (SBRT) enables high dose irradiation of small volumes with a rapid dose fall-off and a limited exposure of nearby healthy structures. The use of SBRT in prostate cancer has been reported by several experiences in the literature, with long-term data that report this technique as a safe and effective treatment for localized prostate cancer [9].

When extreme hypofractionation is adopted, a crucial aspect is represented by target motion management, in order to ensure not only the optimal target coverage, but also the improved sparing of surrounding organs at risk (OARs) [10].

To date, a large amount of data is available about the management of interfraction motion, consisting of the daily assessment of patient preparation in terms of bladder and rectum anatomy, which are the main factors influencing prostate shifts [11, 12].

On the contrary, the investigation of intrafraction motion is less reported in the literature and mainly based on the use of implanted fiducial markers, reporting that major drifts and transitory motions occur mainly in the antero-posterior and cranio-caudal directions [13–15].

Furthermore, although the available imaging consisting of in-room computed tomography proved to be a reliable tool for image guided-radiotherapy (IGRT), in some districts kilovoltage and/or megavoltage CT may offer a limited anatomy visualization due to a sub-optimal soft-tissues contrast [16–18].

Another tool potentially helpful in mitigating prostate motion is represented by rectal hydrogel spacer (SpaceOAR, Boston Scientific,Marlborough, MA, USA), a polyethylene-glycol gel device that is injected in the Denonvilliers fascia pushing forward the prostate in order to enlarge the distance with the anterior rectal wall, by creating an interspace estimated between 7 and 15 mm of extent [19].

In this scenario, the recent introduction of MR-guided linacs may represent a potential game-changer; first, by providing a superior anatomy imaging that allows a better identification of target volumes and OARs structures, also refining contouring accuracy [17]. Secondly, MRI-guidance may improve the quality of inter- and intra-fraction motion assessment. Especially for intrafraction motion, MR-linacs enable a refined evaluation of prostate shifts during treatment by means of pre- and post-treatment MRI acquisitions.

In October 2019 we have started at our department the clinical activity with Unity Elekta MR-linac (Elekta Unity, Stockholm, Sweden). Unity® is a new platform for radiotherapy that conjugates a 7 MV flattening filter free (FFF) linear accelerator with a 1.5 Tesla MR system. In accordance with an ongoing prospective observational study approved by the internal Ethical Committee, we have started MRI-Linac-based treatments also including SBRT for localized prostate cancer [20, 21].

Herein we present our preliminary report on intrafraction prostate motion data registered for the first 20 patients treated with MR-linac, with the aim to assess the potential impact of the use of hydrogel rectal spacer.

## Materials and methods

The following results are derived from the prospective observational study ongoing at our institution, which was approved on April 2019 by the Local Ethical Committee. (MRI/LINAC n°23,748).

Herein we report the data concerning 20 patients who underwent MRI-guided stereotactic body radiotherapy for low-intermediate risk prostate cancer with or without rectal hydrogel spacer placement from October 2019 to January 2020.

Inclusion criteria for prostate SBRT were as follows: age > 18 years, Karnofsky Performance Status> 70, histologically proven low or intermediate risk prostate cancer according to National Comprehensive Cancer Network v.1.2020 classification, no radiological evidence of pathological lymph-nodes or distant metastases, no other malignant tumors in the last 5 years, International Prostate Symptoms Score (IPSS) < 15. Androgen deprivation therapy was allowed according to risk group.

Exclusion criteria were: prostate volume > 80 cc, previous prostate surgery or previous transurethral resection of prostate performed within 6 months from RT, any MRI contraindication (electronic devices, claustrophobia etc.), the inability to gain informed consent.

Hydrogel spacer placement was proposed as optional to all patients, and consisted of a minimally invasive procedure performed under local anesthesia. The Urology Surgeon implanted the spacer in the Denonvilliers fascia, and radiotherapy treatment was scheduled to start within 3 weeks from the surgical procedure.

### Radiotherapy protocol

After consultation, all patients were educated for the imaging protocol which consisted at first of the acquisition of a 3 mm slice thickness pelvis-CT scan in supine position with knee-ankle immobilization devices, for dose calculation purposes. Afterwards, a T2- weighted pelvis MRI was acquired at the Unity MRI-Linac. For both imaging procedures, all patients were required to have a comfortably full bladder (by drinking 500 ml of water before the exam) and an empty rectum (by self-administering a fleet enema).

As far as target volume delineation, in the case of low-risk disease, the clinical target volume (CTV) was the prostate gland alone, while for intermediate risk disease, the entirety of the seminal vesicles was contoured as well. The planning target volume (PTV) was generated by adding a 5 mm margin in all directions, except for the posterior direction where a 3 mm expansion was applied, according to published studies [20, 21].

The rectum, bladder, penile bulb, urethra, and femoral heads were manually contoured as OARs.

The SBRT schedule consisted of five daily fractions of 7 Gy (total prescription dose, *D*_*p*_ = 35 Gy) for all patients, equal to a normalized total dose of 2 Gy per fraction (NTD2) ranging between 70 and 85 Gy, assuming an α/β ratio between 3 and 1.5 Gy for PC.

The dose distribution normalization was calculated to ensure a minimum 95% of the PTV to receive at least the 95% of the prescribed dose, and less than 2% of the PTV to receive 107% of Dp.

For OARs, the following constraints were applied: for the rectum: V18 Gy ≤ 35%, V28 Gy ≤ 10%, V32 Gy ≤ 5%, Dmax ≤35 Gy; for the bladder: Dmax ≤35 Gy; no hotspots for the urethral PRV; for the intestinal loops Dmax < 32 Gy. Dmax was always referred to the hottest 1 cm^3^ of the anatomical structure.

Static field intensity-modulated radiotherapy (IMRT) delivered with 16 beams were applied for generating baseline treatment plans.

Daily adapted radiotherapy is delivered with Unity using two alternative protocols: ‘adapt-to-position’ (ATP) and ‘adapt-to-shape’ (ATS). ATP is based on the daily iso-center position change in reference to the pre-treatment CT. In the case of the ATS workflow, a re-contouring of the daily MRI is performed to adapt the treatment plan to the real-time anatomy of the patient. Thus, ATS allows clinicians to refine the dose delivery based on daily changes in the size, shape, and position of PTV and OARs.

More specifically, before each fraction, a new T2-weighted MRI sequence (preMRI) is acquired and rigidly registered to the simulation MR. The original set of contours is transferred to the daily preMRI using deformable registration, and then edited, where needed, at physician’s discretion. A full re-optimization is performed by the physicist and, during the second optimization phase (i.e. the segmentation phase), a second verification MRI scan is acquired to assess if bladder and rectum deformations can be considered negligible. In the case of unacceptable deformations, the patient was required to repeat the preparation protocol before being repositioned for treatment. Otherwise, the treatment is delivered using a cine-MRI, typically acquired on two coronal and sagittal planes to monitor patient motion. At the end of the session, a further post-MRi scan was performed, to estimate the intra-fraction organ motion.

### Data collection and statistical analysis

A manual re-contouring of the prostate CTV was performed by one physician in both pre- and post-MRI scans. Afterwards, a soft tissue-rigid registration with the daily planning MRI was generated based on the volume of interest (i.e. the prostate CTV). Then, translational and rotational shifts were registered in all directions (cranio-caudal, antero-posterior and latero-lateral) to assess intrafraction motion of the prostate referring to the delivery position.

Descriptive statistics were collected for continuous variables (median, maximum and minimum values and standard deviation). The Mann-Whitney U-test was used to compare the entity of translational and rotational shifts between the spacer and no-spacer cohorts, and the potential impact of prostate CTV volume on organ motion for the entire population. A *p*-value< 0.05 was assumed to be statistically significant. All statistical analyses were performed with Graphpad Prism software v.8.4.2 (Graphpad Software, San Diego, CA, USA).

## Results

The pre- and post-treatment MRI data regarding a total of 100 fractions in 20 consecutive patients who underwent MRI-guided prostate SBRT are herein presented. Ten patients were treated without hydrogel rectal spacer, and ten after the insertion of hydrogel spacer. This procedure was well tolerated in all patients, except in one case who developed rectal tenesmus fully resolved after local steroids. The median time for fraction was 50 min (range, 46–65 min) for the entire cohort and the median CTV volume was 57.3 cc (range, 25.3–74.3 cc). Median spacer interface was 0.99 cm (range, 0.44–1.49 cm). Patients’ characteristics and toxicity patterns are summarized, respectively in Table 1 and Table 2.

**Table 1:** Baseline patients’ characteristics

Age, years (median, range):		
SpaceOAR	70 (54–78)	*p = 0.44*
No-SpaceOAR	66 (56–75)	
**PSA, ng/ml** (median, range):		
SpaceOAR	9.3 (6.6–19)	*p = 0.08*
No-SpaceOAR	6.8 (4.2–12.7)	
**Risk Group** (n / %):		
***(Low/ Favorable Intermediate/Unfavorable Intermediate*)**		
SpaceOAR	3 (30%) / 4 (40%) / 3 (30%)	
No-SpaceOAR	2 (20%) / 6 (60%) / 2 (20%)	
**Androgen deprivation therapy** (n / %):		
SpaceOAR	3 (30%)	
No-SpaceOAR	2 (20%)	
**Prostate Volume, cc** (median, range):		
SpaceOAR	62.5 (49.8–79)	*p = 0.23*
No-SpaceOAR	55.5 (29.7–79)	
**Planning Target Volume, cc** (median, range):		
SpaceOAR	118.8 (85.7–150.1) *p = 0.17*	
No-SpaceOAR	110.3 (70.9–145.3)	
**IPSS score** (median, range):		
SpaceOAR	7 (0–15)s	*p = 0.14*
No-SpaceOAR	5 (0–10)

**Table 2:** Acute Toxicity Rates (CTCAE v.5)

Genitourinary	G2 / G1 (n / %)
-SpaceOAR	2 (20%) / 2 (20%)
-No-SpaceOAR	1 (10%) / 4 (40%)
**Gastrointestinal**	**G2 / G1 (n / %)**
-SpaceOAR	0 (0%) / 1 (10%)
-No-SpaceOAR	0 (0%) / 1 (10%)

In the entire sample of the study, median rotational displacement was 0.1° in cranio-caudal (X-axis), − 0.002° (Y-axis) in latero-lateral and 0.01° (Z-axis) in antero-posterior direction; median translational shift was 0.11 mm in cranio-caudal, − 0.24 mm in latero-lateral and − 0.22 mm in antero-posterior.

For rotational shifts, the median displacement in cranio-caudal direction (X-axis) was 0.18° (range: − 0.37°/0.16°; SD = 0.15°) for the no-spacer subgroup vs 0.11° (range: − 0.70°/0.31; SD = 0.25°) for the spacer subgroup (*p* = 0.108), in latero-lateral direction (Y-axis) we recorded a median displacement of − 0.04° degrees (range: − 0.36°/0.82; SD = 0.33°) in the no-spacer subgroup vs − 0.03° degrees (range: − 0.16°/0.13°; SD = 0.08°) in the spacer subgroup (*p* = 0.78). A statistically significant difference was observed in the antero-posterior direction (Z-axis) between the spacer and the no-spacer cohorts (*p* = 0.033), with respective values of − 0.0005° (range: − 0.30°/0.12; SD = 0.11°) and 0.09° (range: − 0.08°/0.26°; SD = 0.10°).

**Fig. 1:**
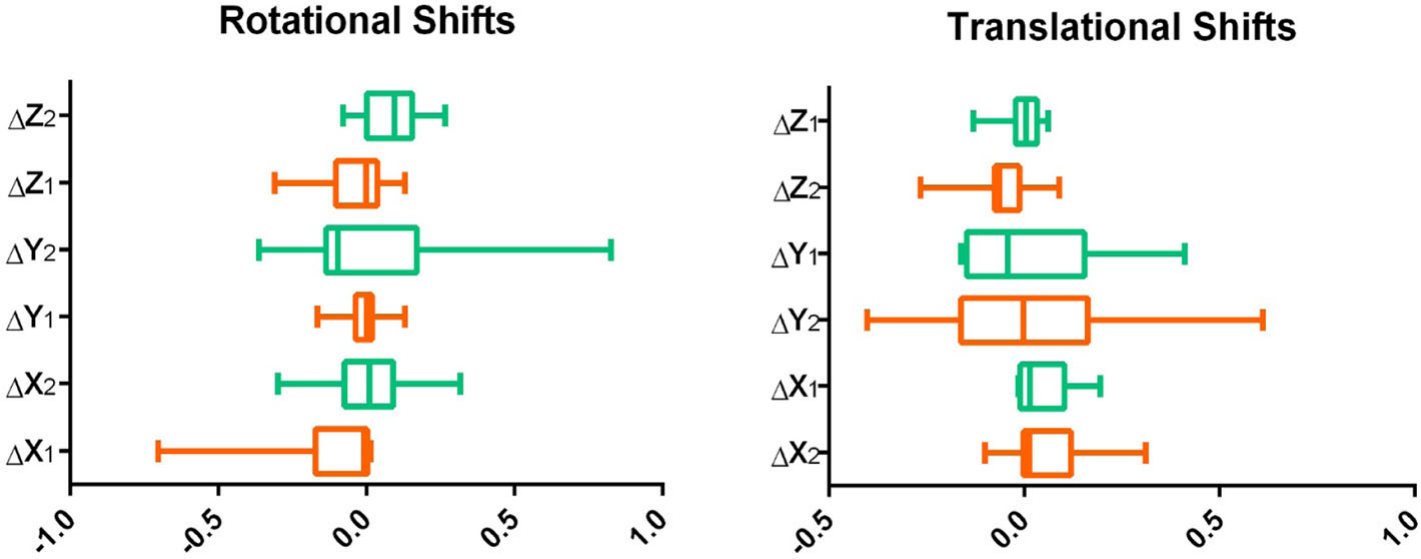
Box plot graphics of rotational and translational shifts in all directions in patients with (1-orange) and without spacer (2-turquoise)

As far as translational shifts, in cranio-caudal direction we recorded a median displacement of respectively 0.06 mm (range: − 1.07 mm/0.89 mm; SD = 0.46 mm) and − 0.42 mm (range: − 0.16 mm/3.1 mm; SD = 1.25 mm) in the no-spacer and spacer patients (*p* = 0.75), while for latero-lateral direction median displacement values were − 0.15 mm (range: − 2.5 mm/4.1 mm; SD = 2.5 mm) and − 0.24 mm (range: − 1.42 mm/4.5 mm; SD = 2.5 mm) for spacer and no-spacer cohorts (*p* = 0.77). In the Z-axis, the spacer subgroup reported a translational shift of − 0.42 mm (range:-1.34 mm/0.89 mm; SD = 0.67 mm) vs − 0.17 mm (range: − 2.65 mm/0.60 mm; SD = 0.91 mm) in the no-spacer subgroup (*p* = 0.07). (Table 3 and Fig. 1).

**Table 3:** Rotational and translational shifts with and without spacer

	Spacer			No Spacer			*p-*value
	Median	SD	Range	Median	SD	Range	
Rotational shifts (degrees)							
AP	−0.0005°	0.11°	−0.30°/0.12°	0.09°	0.10°	−0.08°/0.26°	0.033
LL	−0.03°	0.08°	−0.16°/0.13°	−0.04°	0.33°	−0.36°/0.82°	0.78
CC	0.11°	0.25°	−0.70°/0.31	0.18°	0.15°	−0.37°/0.168°	0.108
Translational shifts (mm)							
AP	−0.42	0.67	−1.34/0.89	−0.17	0.91	−2.65/0.6	0.07
LL	−0.15	2.5	−2.5/4.1	−0.24	2.5	−1.42/4.5	0.77
CC	−0.42	1.25	−0.16/3.1	0.06	0.46	−1.07/0.89	0.75

*AP* Antero-posterior, *CC* Cranio-caudal, *LL* Latero-lateral, *SD* Standard deviation

**Fig. 2:**
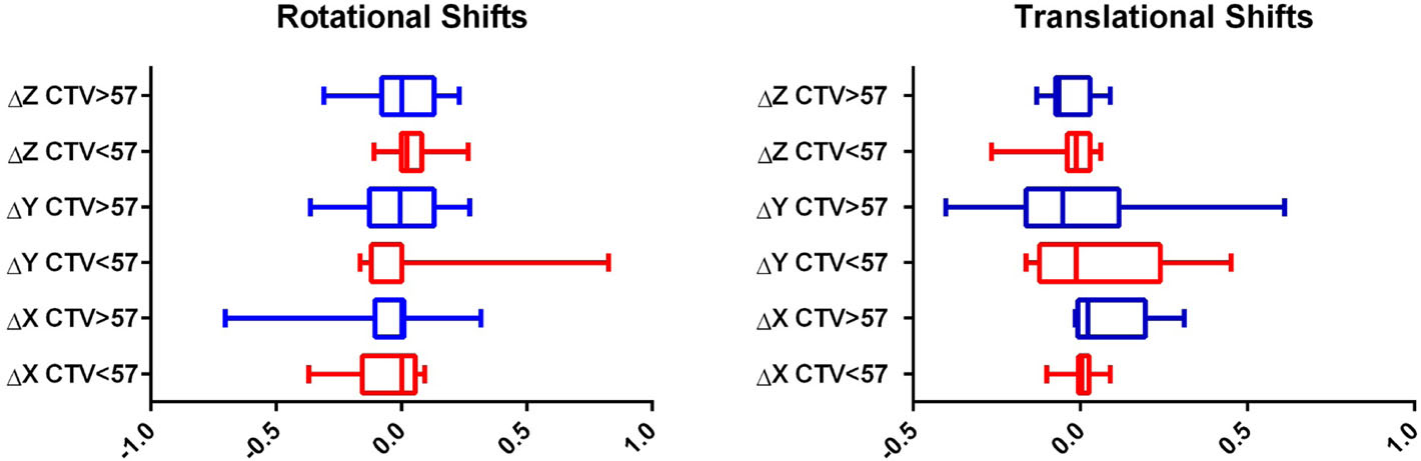
Box plot graphics of rotational and translational shifts according to prostate CTV volume > 57 cc (blue) or < 57 cc (red)

We have also investigated the potential impact of prostate CTV volume on organ motion, reporting no statistically significant correlation when comparing rotational and translational shifts of patients with a prostate CTV > 57.3 cc or < 57.3 cc. (Table 4 and Fig. 2).

**Table 4:** impact of prostate CTV volume on rotational and translational shifts

	CTV > 57.3 cc or CTV < 57.3 cc
	*p-*value
Rotational shifts	
AP	0.98
LL	0.69
CC	0.78
Translational Shifts	
AP	0.46
LL	0.61
CC	0.26

As far as the dosimetric analysis, we observed statistically significant differences in the pre-treatment planning phase in favor of the spacer cohort for several rectum dose constraints: rectum V32Gy < 5% (*p* = 0.001), V28 Gy < 10% (*p* = 0.001) and V18Gy < 35% (*p* = 0.039). Also for bladder V35 Gy < 1 cc, the use of spacer provided a dosimetric advantage compared to the no-spacer subpopulation (*p* = 0.04). Furthermore, PTV V33.2Gy > 95% was higher in the spacer cohort compared to the no-spacer one (*p* = 0.036).

As far as the daily adapted planning, the use of rectal spacer was found to produce a dosimetric advantage in terms of rectum sparing and PTV coverage. More specifically, all the dose constraints used for rectum were significantly reduced as follows: rectum V35Gy < 1 cc (*p* = 0.0001), V32Gy < 5% (*p* = 0.00001), V28 Gy < 10% (*p* < 0.00001), V18Gy < 35% (*p* < 0.00001). Similarly to the baseline planning phase, bladder V35 Gy < 1 cc was found to be reduced in the spacer cohort (*p* = 0.00004). Notably, also the planning goals for tumor coverage (i.e. PTV V33.2Gy > 95% and PTV 37.5 Gy < 2%) were improved in the spacer group vs the no-spacer one (*p* = 0.002 and *p* = 0.0001, respectively). (Table 5).

**Table 5:** Baseline planning and daily adaptive treatment sessions dosimetric data in patients with and without spacer

Dosimetric Parameters	SpaceOAR group (mean ± SD)	No-SpaceOAR group (mean ± SD)	p
**Baseline Planning:**			
Rectum			
V35Gy < 1 cc	0.01 ± 0.02	0.07 ± 0.14	0.21
V32Gy < 5%	0.73 ± 0.6	3.28 ± 0.9	*0.001*
V28Gy < 10%	2.48 ± 1.73	7.88 ± 0.98	*0.001*
V18Gy < 35%	15.99 ± 4.53	19.98 ± 2	*0.039*
Rectal Volume (cc)	72.6 ± 39.8	46 ± 8	0.06
Bladder			
V35Gy < 1 cc	0.09 ± 0.11	0.24 ± 0.21	*0.04*
V30Gy (%)	7.09 ± 4.90	8.15 ± 3.42	0.24
V20Gy (%)	17.19 ± 9.41	19.7 ± 6.74	0.21
V10Gy (%)	36.25 ± 12.89	39.86 ± 11.35	0.43
V5Gy (%)	46.98 ± 16.58	54.85 ± 16.22	0.21
Bladder Volume (cc)	390.9 ± 182.8	255.6 ± 108.8	0.06
Urethra			
V35 Gy < 1 cc	0.3 ± 0.31	0.33 ± 0.25	0.72
V33.2 Gy > 95%	100 ± 0	99.94% ± 0.18%	0.35
PTV			
PTV37.5Gy < 2%	0.5 ± 0.62	0.74 ± 0.67	0.35
PTV33.2Gy > 95%	98.55 ± 1.11	96.8% ± 1.25%	*0.036*
PTV Volume (cc)	121.8 ± 20.9	106.5 ± 27	0.17
**Adaptive daily Planning:**			
Rectum			
V35Gy < 1 cc	0.01 ± 0.03	0.10 ± 0.16	*0.0001*
V32Gy < 5%	0.88 ± 1	3.43 ± 1.33	*0.00001*
V28Gy < 10%	2.95 ± 2.46	8.07 ± 1.61	*0.00001*
V18Gy < 35%	14.97 ± 5.76	20.81 ± 2.59	*0.00001*
Rectal Volume (cc)	49.7 ± 16.2	47.7 ± 10	0.16
Bladder			
V35Gy < 1 cc	0.04 ± 0.09	0.25 ± 0.32	*0.00004*
V30Gy (%)	6.98 ± 2.79	7.67 ± 5.86	0.43
V20Gy (%)	18.75 ± 6.86	19.33 ± 10.41	0.78
V10Gy (%)	40.06 ± 13.17	39.18 ± 14.85	0.70
V5Gy (%)	52.97 ± 17.77	53.37 ± 19.97	0.83
Bladder Volume (cc)	215.4 ± 166.9	217.5 ± 112.5	0.17
Urethra			
V35 Gy < 1 cc	0.23 ± 0.20	0.22 ± 0.17	0.92
V33.2 Gy > 95%	99.98 ± 0.13	100% ± 0%	0.29
PTV			
PTV37.5Gy < 2%	0.45 ± 0.51	1.39 ± 1.05	*0.0001*
PTV33.2Gy > 95%	97.91 ± 1.35	95.96% ± 4.01%	*0.002*
PTV Volume (cc)	118.3 ± 40.1	111.4 ± 35.5	*0.12*

## Discussion

The present paper depicts preliminary data of a total of 100 fractions in a series of 20 patients treated with MR-guided SBRT for prostate cancer, including in the sample 10 patients who received radiotherapy after the insertion of a rectal hydrogel spacer and 10 patients who were treated without spacer.

The implementation of rectal spacer in prostate SBRT is known to have a favorable impact on minimizing the dose to the anterior rectal wall, despite few data are currently available in terms of estimates of prostate movements during radiotherapy [22, 23].

Peri-rectal hydrogel spacer consists of a layer of polyethylene-glycol gel (SpaceOAR, Boston Scientific, Marlborough, MA, USA) implanted in the Denonvilliers’ fascia in order to outdistance the prostate from anterior rectal wall. At the same time, this device is presumed to limit organ motion in the Z-axis, providing a sort of stabilization effect of the gland. To date, there is no clear evidence supporting the use of rectal spacer as a means to fixate prostate gland for the intrafraction motion, as some Authors hypothesize a potentially detrimental rectal wall inflammation [24].

Hedrick et al. reported in a series of 41 patients treated with proton therapy for prostate cancer with hydrogel spacer a significant advantage in terms of rectal sparing when compared to the dosimetric advantages provided by the use of endorectal balloons [25].

In agreement, in a previous report of our institution with conventional Linacs, we have also observed a dosimetric advantage provided by rectal gel in terms of anterior rectal wall exposure to low doses. Notably, in the abovementioned previous experience, the gel also improved the PTV coverage in terms of V95% [19].

These advantages have also been recorded in the present experience in further support of the use of the hydrogel spacer in terms of superior rectal sparing and increased PTV coverage. Nonetheless, these data are preliminary and a longer follow-up is required to assess any potential impact on clinical outcomes and toxicity reports.

The role of hydrogel spacer on prostate motion patterns has been investigated in few previous experiences in the literature. Juneja et al. [26] reported in their series of 26 patients (respectively 12 with and 14 without spacer) a mean motion> 3 mm only in the 5% of delivered fractions, including also hypofractionated courses, while in our series we observed > 3 mm shifts in 2 cases (one in the X- and one in the Z-axis) among the spacer subgroup versus 3 cases in the no-spacer subgroup, where > 3 mm shifts were detected entirely in the latero-lateral direction. In another study, Picardi et al. [27] recorded in a cohort of 20 patients, treated with or without spacer, a displacement > 5 mm in latero-lateral, cranio-caudal and antero-posterior direction respectively in the 0.8, 6.5 and 12.5% of cases, still not reaching a statistically significant difference between the two cohorts, unlike our series.

Similarly Pinkawa et al. [28] recorded a favorable effect provided by the use of rectal spacer not only in terms of outdistancing the prostate from the anterior rectal wall, but also in reducing larger posterior displacements when compared to patients treated without spacer insertion.

Finally, in a case report by Sumila et al. [29], a Cyberknife treatment for prostate SBRT recorded, over all the 5 treatment sessions, prostate shifts within 4 mm in all directions, regardless of the one hour-long treatment time. Despite referring to a single patient, this feature seems to support the stabilization effect on prostate motion, even in the case of longer treatment time techniques.

To the best of our knowledge, the present paper is the first analysis of prostate intra-fraction motion in MR-guided SBRT comparing the outcomes of patients treated with or without rectal hydrogel spacer. Keeping in mind the small sample size of the present study, we hypothesize that the combination of MR-guided SBRT and the use of rectal hydrogel spacer may favorably impact on stereotactic radiotherapy for prostate cancer. To date, we have no such longer follow-up to draw definitive conclusions on this matter, but it will surely be analyzed with larger data. Nonetheless, we believe that the combination of MRI-Linac and the use of SpaceOar hydrogel is feasible and well tolerated from patients, as reported in a previous report in which we have recorded no significant differences in terms of QoL between baseline and post-RT scores [21].

Despite being preliminary data, in our series we have observed a favorable impact of the use of hydrogel spacer in antero-posterior rotational and translational shifts, reporting a statistically significant impact on minimizing prostate displacement in the rotational antero-posterior direction (*p* = 0.033) and a positive trend for the antero-posterior translational shift (*p* = 0.07), although not reaching a statistically significant value.

The higher motion in the antero-posterior direction has also been reported in a fiducial markers-guided study by Kotte et al. [30] observing an intrafraction motion > 2 mm in radiotherapy sessions of 5–7 min in 66% of cases. Consequently, the Authors recommended a minimum 2 mm margin to keep into account the intrafraction motion, but larger margins are still required to cover any other potential uncertainty, as also described by other studies of prostate motion based on the use of fiducial markers [31].

Compared to other experiences investigating the role of spacer by means of Cone Beam CT imaging, MRI-Linacs provide a superior visualization of the patient’s anatomy enabling clinicians to improve the accuracy in target volume delineation and to better distinguish the nearby healthy structures [32]. Furthermore, the adaptive workflow based on the daily re-planning based on the real-time anatomy refines the delivery process. Notably, these advantages may be theoretically counterbalanced by the longer treatment time for session, even if the potential impact of treatment time on organ motion still remains a matter of debate [33].

Nonetheless, the improved accuracy in real-time anatomy visualization along with a careful assessment of organ motion during the beam-on-time will be major assumptions to determine the proper margin expansion for radiotherapy treatment [34, 35], as in our series we recorded prostate displacement variation still within the expansion protocol of our Institution.

## Conclusion

In the present study, the application of peri-rectal hydrogel spacer for prostate SBRT resulted in a statistically significant impact on rotational antero-posterior shifts compared to no-spacer cohort, contributing to limit prostate intra-fraction motion. Further studies with larger sample size and longer follow-up are required to confirm this ideally favorable effect and to assess any potential impact on clinical outcomes.

## Data Availability

Not applicable.

## Abbreviations

ATP: Adapt-to-position
ATS: Adapt-to-shape
CT: Computed tomography
CTV: Clinical target volume
FFF: Flattening filter free
IGRT: Image guided radiotherapy
IMRT: Intensity modulated radiotherapy
IPSS: International prostate symptom score
MRI: Magnetic resonance imaging
NTD2: Normalized total dose at 2 Gy per fraction
OAR: Organ at risk
PC: Prostate cancer
PRV: Planning organ at risk volume
PTV: Planning target volume
SBRT: Stereotactic body radiotherapy
SD: Standard deviation
